# Towards hypernuclei from nuclear lattice effective field theory

**DOI:** 10.1140/epja/s10050-024-01427-y

**Published:** 2024-10-30

**Authors:** Fabian Hildenbrand, Serdar Elhatisari, Zhengxue Ren, Ulf-G. Meißner

**Affiliations:** 1 Institute for Advanced Simulation, Forschungszentrum Jülich, 52425 Jülich, Germany; 2 Faculty of Natural Sciences and Engineering, Gaziantep Islam Science and Technology University, 27010 Gaziantep, Turkey; 3 Department of Physics, King Fahd University of Petroleum & Minerals, Dhahran, 31261 Saudi Arabia; 4 Helmholtz-Institut für Strahlen- und Kernphysik and Bethe Center for Theoretical Physics, Universität Bonn, 53115 Bonn, Germany

## Abstract

Understanding the strong interactions within baryonic systems beyond the up and down quark sector is pivotal for a comprehensive description of nuclear forces. This study explores the interactions involving hyperons, particularly the $$\varLambda $$ particle, within the framework of nuclear lattice effective field theory (NLEFT). By incorporating $$\varLambda $$ hyperons into the NLEFT framework, we extend our investigation into the $$S = -1$$ sector, allowing us to probe the third dimension of the nuclear chart. We calculate the $$\varLambda $$ separation energies ($$B_{\varLambda }$$) of hypernuclei up to the medium-mass region, providing valuable insights into hyperon–nucleon (*YN*) and hyperon–nucleon–nucleon (*YNN*) interactions. Our calculations employ high-fidelity chiral interactions at N$$ ^3$$LO for nucleons and extend it to $$\varLambda $$ hyperons with leading-order S-wave *YN* interactions as well as *YNN* forces constrained only by the $$A=4,5$$ systems. Our results contribute to a deeper understanding of the SU(3) symmetry breaking and establish a foundation for future improvements in hypernuclear calculations.

## Introduction

Understanding the strong interactions in the light quark sector is crucial for a comprehensive description of baryonic systems. In the up and down quark (nucleonic - *N*) sector, the strong interactions can be described by highly successful phenomenological potential models based on meson field theory and dispersion relations, such as Paris *NN* [1], Stony Brook *NN* [2], Nijmegen I-II *NN* [3], AV18 *NN* [4], CD Bonn *NN* [5], Urbana-Argon *NN* and 3*N* [6], and Urbana-Illinois 3*N* [7] potentials or by the framework of chiral effective field theory ($\chi$EFT) [8, 9] which is considered the modern theory of the nuclear forces between nucleons. To probe the strong interactions beyond the up and down quark sector, hyperons, such as the $\varLambda$ particle, offer a unique opportunity by extending the traditional nuclear chart into the third dimension, combining a hyperon with a nucleus to form hypernuclei. Since the Pauli exclusion principle does not apply between nucleons and hyperons, in this new type of atomic nuclei the $\varLambda$ separation energy, $B_{\varLambda }$, can exceed the binding energy per nucleon in conventional nuclear systems, although the latter is larger in light hypernuclei.

The study of hypernuclei provides valuable insights into the baryon–baryon interactions, and an accurate description of the properties of hypernuclei requires a systematic formulation of interactions between hyperons and nucleons, as well as constraining their low-energy constants (LECs). The great success of both phenomenological potential models and chiral EFT for nucleons is based on rich and precise *NN*-scattering data and nuclear binding energies. However, due to the scarcity of hyperon–nucleon and hyperon–hyperon scattering data, the spectra of hypernuclei are pivotal in constraining these interactions, deepening our understanding of SU(3) flavor symmetry breaking and charge symmetry breaking in strong interactions.

There has been intense interest and significant progress in the study of hypernuclei from both theoretical and experimental programs. For a comprehensive review of past and recent efforts, see, for example, Ref. [10]. Early theoretical work on medium-mass hypernuclei has been explored using the shell model [11, 12] and phenomenological models [13–15]. Calculations for larger hypernuclear systems typically employ the *G*-matrix method and Skyrme Hartree-Fock approaches [16–23], as well as relativistic mean-field models [24–27]

One of the leading theoretical efforts to investigate hypernuclei is the No-Core-Shell-Model (NCSM), which describes light hypernuclei with great precision using interactions derived within $\chi$EFT [28–34]. However, this method is currently not suited for addressing the medium- and heavy-mass regions due to computational scaling. Light hypernuclei have also been explored using cluster models [35–37]. Additionally, the light mass region has been studied within the framework of pionless effective field theory [38–41]. Furthermore, initial studies of hypernuclear systems using quantum Monte Carlo (MC) calculations have been performed in the medium and heavy mass regions [42, 43].

Experimentally, hypernuclei are often produced through reactions like ($K^-$, $\pi ^-$) [44–46], ($\pi ^+$, $K^+$) [47–49], and electromagnetic processes ($e,e^{\prime }K^+$) [50, 51]. Early experiments using ion collisions at GeV energies demonstrated the feasibility of producing light hypernuclei [52], with subsequent experiments at facilities like Dubna [53] and GSI [54, 55] further advancing the field. Modern experiments at J-PARC [56, 57] and the ALICE experiment at LHC [58, 59] as well as BESIII [60, 61] continue to investigate hyperon–nucleon interactions.

In this work, we study hypernuclei system in the framework of nuclear lattice effective field theory (NLEFT). NLEFT is a powerful quantum many-body method that combines the advantages of effective field theories with lattice methods [62, 63]. It offers the unique feature that the computational time scales only with the mass number *A*. The method has recently been used to compute the ground state energies, excited state energies and charge radii of light and medium-mass nuclei, and to describe the saturation energy and density of symmetric nuclear matter at next-to-next-to-next-to-leading order (N$^3$LO) in $\chi$EFT simultaneously reproducing accurate two-nucleon phase shifts and mixing angles [64].

In the framework of NLEFT, the inclusion of $\varLambda$ hyperons was first explored in Ref. [65] using the impurity lattice Monte Carlo (ILMC) method [66]. This study employed a simplified Wigner SU(4)-symmetric interaction to compute the binding energies of light hypernuclei $^3_\varLambda$H, $^4_\varLambda$H and $^5_\varLambda$He. The ILMC method treats single $\varLambda$ hyperons as worldlines in a medium of nucleons simulated by the Auxiliary Field Quantum Monte Carlo (AFQMC) method. Later, the ILMC method was expanded to the study of systems containing multiple hyperons [67]. Recently, a novel AFQMC approach has been introduced to enable the efficient calculations of hypernuclear systems with an arbitrary number of hyperons, and lattice simulations for pure neutron matter and hyper-neutron matter up to five times nuclear matter saturation density have been performed [68].

In this paper, we extend the ab initio method reported in [62–64] towards the $S=-1$ sector by including the lightest hyperon, the $\varLambda$, and its interactions with nucleons. This allows us to address the third dimension of the nuclear chart within this powerful framework. As a starting point we calculate $\varLambda$ separation energies of hypernuclei up to the medium-mass region in the framework of NLEFT. This work not only advances our theoretical understanding but also lays the groundwork for future improvements in hypernuclear calculations, ultimately contributing to a deeper comprehension of the strong interaction in baryonic systems. Possible applications in the future include an in depth structure analysis of hypernuclei using the pinhole algorithm similar to the work done for the carbon nucleus in Ref. [69].

The paper is structured in the following way. We start with a brief overview about the general formalism and the underlying interactions in Sect. 2, at which point we introduce the newly included *YN* and *YNN* forces, before discussing the fitting procedure of the latter ones in Sect. 2.2. After a brief discussion of finite box size effects in Sect. 2.3, we conclude this paper with a detailed presentation and analysis of a selection of medium-mass hypernuclei with $A = 3 \ldots 16$ in Sect. 3. Finally, we discuss potential improvements to the methods used here. Technical details are relegated to the appendices.

## Lattice Hamiltonian

**Fig. 1:**
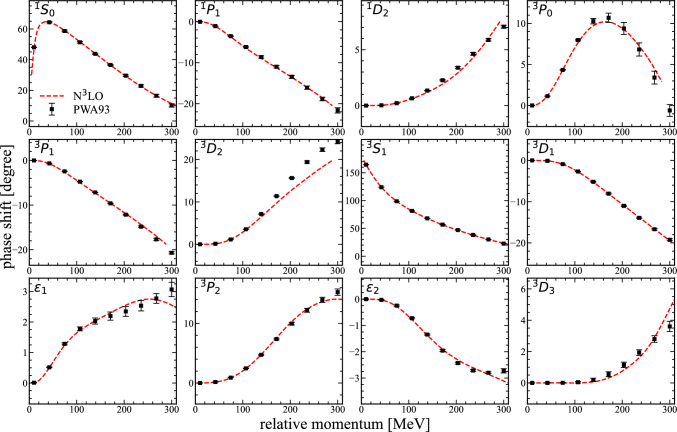
The neutron-proton scattering phase shifts (S, P, D waves) and mixing angles ($\epsilon _{1,2}$) up to N$^3$LO in $\chi$EFT versus the relative momentum. The partial wave analysis is taken from Ref. [73]

We start by constructing and discussing the lattice Hamiltonian needed for systems consisting of nucleons and $\varLambda$ hyperons. In general, two different types of interactions can be defined, the ones involving only nucleons and the interactions containing additional hyperons. For the former, we use high-fidelity chiral interaction at N$^3$LO and the quantum many-body approach, so called wave function matching, developed in Ref. [64]. This lattice action has been used to successfully describe the spectrum and charge radii of nuclei over a large part of the nuclear chart as well as the saturation properties of nuclear matter [64], structure factors for hot neutron matter [70], and the nuclear charge radii of silicon isotopes [71] simultaneously maintaining accurate two-nucleon phase shifts and mixing angles as shown in Fig. 1. Since the lattice operators and lattice Hamiltonian for nucleons are extensively discussed in the Supplementary Sections of Ref. [64], we will focus primarily on the lattice operators and interactions involving hyperons.

For the latter ones, in this work, we limit ourselves to leading order S-wave $\varLambda N$ contact interactions:1and additional contact three-body $\varLambda NN$ forces as derived in Ref. [72] 2a2b2c where $\varvec{\tau },\varvec{\sigma }$ are Pauli-(iso)spin matrices and $C_i$ are the respective LECs. The three-body forces (TBFs) appear at N$^2$LO in the chiral power counting à la Weinberg. In pionless EFT the three-body forces, however, would be leading order. Since here we do not consider the explicit two-pion exchange interactions, which are effectively simulated by the smearing discussed below, we promote the three-baryon forces in the $S=-1$ sector to LO. Note further that the one-pion exchange is suppressed for the $\varLambda N$ interaction due to isospin symmetry and hence not part of this effective leading order interaction. Finally, due to the fact that high-fidelity chiral interactions between nucleons are well tested, those set the smearing parameters as well as the lattice spacing for the hypernuclear interactions. Therefore, our lattice Hamiltonian is defined as,

3 $$\begin{aligned} H = H_\mathrm{N^3LO} + H^{Y}_\textrm{free} + V_{Y N} + V_{YNN}\,, \end{aligned}$$

where $H_\mathrm{N^3LO}$ is the high-fidelity Hamiltonian for nucleons [64], $H^{\varLambda }_\textrm{free}$ is the kinetic energy term for $\varLambda$ hyperons defined by using fast Fourier transforms to produce the exact dispersion relations $E_\varLambda =p^2/(2m_{\varLambda })$ with hyperon mass $m_{\varLambda }=1115.68$ MeV, $V_{Y N}$ and $V_{YNN}$ are the hyperon–nucleon and hyperon–nucleon–nucleon interactions given in Eqs. (1) and (2), respectively.

Before describing $$V_{Y N}$$ and $$V_{YNN}$$ interactions, we define the total densities for nucleons and hyperons. We begin with the non-smeared total nucleon, spin and isospin densities at lattice side $${\varvec{n}}$$ in terms of annihilation (creation) operators $$a_{i,j} $$ ($$a_{i,j}^{\dagger }$$),

4a $$\begin{aligned} \rho ({\varvec{n}})=&\sum _{i,j=0,1}a_{i,j}^\dagger ({\varvec{n}})a_{i,j}  ({\varvec{n}})\,, \end{aligned}$$

4b $$\begin{aligned} \rho _S({\varvec{n}})=&\sum _{i,j,i^{\prime }=0,1} a_{i,j}^\dagger ({\varvec{n}}) [\varvec{\sigma }_{S}]_{i,i^{\prime }}a_{i^{\prime },j}  {({\varvec{n}})} \end{aligned}$$

4c $$\begin{aligned} \rho _I({\varvec{n}}) =&\sum _{i,j,j^{\prime }=0,1} a_{i,j}^\dagger ({\varvec{n}}) [\varvec{\tau }_{I}]_{j,j^{\prime }}a_{i,j^{\prime }}  {({\varvec{n}})} \end{aligned}$$

4d $$\begin{aligned} \rho _{SI}({\varvec{n}}) =&\sum _{i,j,i^{\prime },j^{\prime }=0,1} a_{i,j}^\dagger ({\varvec{n}}) [\varvec{\sigma }_{S}]_{i,i^{\prime }}[\varvec{\tau }_{I}]_{j,j^{\prime }}a_{i^{\prime },j^{\prime }}({\varvec{n}})\,, \end{aligned}$$

and the non-smeared total hyperon and spin densities in terms of annihilation (creation) operators $$b_{i} $$ ($$b_{i}^{\dagger }$$),

5a $$\begin{aligned} \xi ({\varvec{n}}) =&\sum _{i=0,1} b_{i}^\dagger ({\varvec{n}})b_{i}  ({\varvec{n}})\,, \end{aligned}$$

5b $$\begin{aligned} \xi _{S}({\varvec{n}}) =&\sum _{i,i^{\prime }=0,1} b_{i}^\dagger ({\varvec{n}}) [\varvec{\sigma }_{S}]_{i,i^{\prime }} b_{i^{\prime }} ({\varvec{n}})\,, \end{aligned}$$

where $i = 0, 1$ (up, down) denotes the spin index, and $j = 0, 1$ (proton, neutron) is the isospin index. Similarly, we define the non-locally smeared operators with $\varvec{\tau }_I$, $\varvec{\sigma }_S$ the Pauli-matrices in isospin- and spin-space, respectively,

6a $$\begin{aligned} {\hat{\rho }}({\varvec{n}})&=\sum _{i,j=0,1}{\tilde{a}}_{i,j}^\dagger ({\varvec{n}}) {\tilde{a}}_{i,j}  ({\varvec{n}})\,, \end{aligned}$$

6b $$\begin{aligned} {\hat{\rho }}_S({\varvec{n}})&=\sum _{i,j,i^{\prime }=0,1} {\tilde{a}}_{i,j}^\dagger ({\varvec{n}}) [\varvec{\sigma }_{S}]_{i,i^{\prime }}{\tilde{a}}_{i^{\prime },j}  ({\varvec{n}})\,, \end{aligned}$$

6c $$\begin{aligned} {\hat{\rho }}_I({\varvec{n}})&=\sum _{i,j,j^{\prime }=0,1} {\tilde{a}}_{i,j}^\dagger ({\varvec{n}}) [\varvec{\tau }_{I}]_{j,j^{\prime }}{\tilde{a}}_{i,j^{\prime }}  {({\varvec{n}})} \,, \end{aligned}$$

6d $$\begin{aligned} {\hat{\rho }}_{SI}({\varvec{n}})&=\sum _{i,j,i^{\prime },j^{\prime }=0,1} {\tilde{a}}_{i,j}^\dagger ({\varvec{n}}) [\varvec{\sigma }_{S}]_{i,i^{\prime }}[\varvec{\tau }_{I}]_{j,j^{\prime }}{\tilde{a}}_{i^{\prime },j^{\prime }}  ({\varvec{n}})\,, \end{aligned}$$

6e $$\begin{aligned} {\hat{\xi }}({\varvec{n}})&= \sum _{i=0,1} {\tilde{b}}_{i}^\dagger ({\varvec{n}}) {\tilde{b}}_{i}  ({\varvec{n}})\,, \end{aligned}$$

6f $$\begin{aligned} {\hat{\xi }}_{S}({\varvec{n}})&= \sum _{i,i^{\prime }=0,1} {\tilde{b}}_{i}^\dagger ({\varvec{n}}) [\varvec{\sigma }_{S}]_{i,i^{\prime }} {\tilde{b}}_{i^{\prime }}  ({\varvec{n}})\,, \end{aligned}$$

where ${\tilde{a}}$ (${\tilde{a}}^{\dagger }$) is the non-locally smeared annihilation (creation) operator for nucleons,

7 $$\begin{aligned} {\tilde{a}}_{i,j}({\varvec{n}})=a_{i,j}({\varvec{n}})+s_\textrm{NL}\sum _{|{\varvec{n}}^{\prime }-{\varvec{n}}|=1}a_{i,j}({\varvec{n}}^{\prime }). \end{aligned}$$

and ${\tilde{b}}$ (${\tilde{b}}^{\dagger }$) is the non-locally smeared annihilation (creation) operator for hyperons,

8 $$\begin{aligned} {\tilde{b}}_{i}({\varvec{n}})= b_{i}({\varvec{n}}) + s_\textrm{NL}\sum _{|{\varvec{n}}^{\prime }-{\varvec{n}}|=1} b_{i}({\varvec{n}}^{\prime }), \end{aligned}$$

with the non-local smearing parameter $s_\textrm{NL}$. We now define the purely locally smeared density operators with a local smearing parameter $s_\textrm{L}$ and a range *d* as,

[3]

9a $$\begin{aligned} \rho ^{(d)}({\varvec{n}})&= \sum _{i,j=0,1} a^{\dagger }_{i,j}({\varvec{n}}) \, a_{i,j}({\varvec{n}}) \nonumber \\&\quad + s_\textrm{L} \sum _{|{\varvec{n}}-{\varvec{n}}^{\prime }|^2 = 1}^d \, \sum _{i,j=0,1} a^{\dagger }_{i,j}({\varvec{n}}^{\prime }) \, a_{i,j}({\varvec{n}}^{\prime }) \,,\end{aligned}$$

9b $$\begin{aligned} \rho ^{(d)}_{S}({\varvec{n}}) =&\sum _{i,j,i^{\prime }=0,1} a^{\dagger }_{i,j}({\varvec{n}}) \, [ {\varvec{\sigma }}_{S}]_{i,i^{\prime }} \, a_{i^{\prime },j}({\varvec{n}}) \nonumber \\&\quad + s_\textrm{L} \sum _{|{\varvec{n}}-{\varvec{n}}^{\prime }|^2 = 1}^d \sum _{i,j,i^{\prime }=0,1} a^{\dagger }_{i,j}({\varvec{n}}^{\prime }) \, [ {\varvec{\sigma }}_{S}]_{ii^{\prime }} \, a_{i^{\prime },j}({\varvec{n}}^{\prime }) \,, \end{aligned}$$

9c $$\begin{aligned} \rho ^{(d)}_{I}({\varvec{n}})&= \sum _{i,j,j^{\prime }=0,1} a^{\dagger }_{i,j}({\varvec{n}}) \, [ {\varvec{\tau }}_{I}]_{j,j^{\prime }} \, a_{i,j^{\prime }}({\varvec{n}}) \nonumber \\&\quad + s_\textrm{L} \sum _{|{\varvec{n}}-{\varvec{n}}^{\prime }|^2 = 1}^d \, \sum _{i,j,j^{\prime }=0,1} a^{\dagger }_{i,j}({\varvec{n}}^{\prime }) \, [ {\varvec{\tau }}_{I}]_{jj^{\prime }} \, a_{i,j^{\prime }}({\varvec{n}}^{\prime }) \,, \end{aligned}$$

9d $$\begin{aligned} \rho ^{(d)}_{SI}({\varvec{n}})&= \sum _{i,j,i^{\prime },j^{\prime }=0,1} a^{\dagger }_{i,j}({\varvec{n}}) \, [ {\varvec{\sigma }}_{S}]_{ii^{\prime }} \, [ {\varvec{\tau }}_{I}]_{j,j^{\prime }} \, a_{i^{\prime },j^{\prime }}({\varvec{n}}) \nonumber \\&\quad + s_\textrm{L} \sum _{|{\varvec{n}}-{\varvec{n}}^{\prime }|^2 = 1}^d \, \sum _{i,j,i^{\prime },j^{\prime }=0,1} a^{\dagger }_{i,j}({\varvec{n}}^{\prime }) [ {\varvec{\sigma }}_{S}]_{ii^{\prime }} \, [ {\varvec{\tau }}_{I}]_{j,j^{\prime }} \, a_{i,j^{\prime }}({\varvec{n}}^{\prime }) \,, \end{aligned}$$

9e $$\begin{aligned} \xi ^{(d)}({\varvec{n}})&= \sum _{i=0,1} b^{\dagger }_{i}({\varvec{n}}) \, b_{i}({\varvec{n}}) \hspace{3.5cm} \nonumber \\&\quad + s_\textrm{L} \sum _{|{\varvec{n}}-{\varvec{n}}^{\prime }|^2 = 1}^d \, \sum _{i,j=0,1} b^{\dagger }_{i}({\varvec{n}}^{\prime }) \, b_{i}({\varvec{n}}^{\prime })\,, \end{aligned}$$

9f $$\begin{aligned} \xi ^{(d)}_{S}({\varvec{n}})&= \sum _{i,i^{\prime }=0,1} b^{\dagger }_{i}({\varvec{n}}) \, [ {\varvec{\sigma }}_{S}]_{i,i^{\prime }} \, b_{i^{\prime }}({\varvec{n}}) \nonumber \\&\quad + s_\textrm{L} \sum _{|{\varvec{n}}-{\varvec{n}}^{\prime }|^2 = 1}^d \, \sum _{i,i^{\prime }=0,1} b^{\dagger }_{i}({\varvec{n}}^{\prime }) \, [ {\varvec{\sigma }}_{S}]_{i,i^{\prime }} \, b_{i^{\prime }}({\varvec{n}}^{\prime }) \,. \end{aligned}$$

Finally, we define both locally and non-locally smeared density operators as,

10a $$\begin{aligned} {\tilde{\rho }}({\varvec{n}})&= \sum _{i,j=0,1} {\tilde{a}}^{\dagger }_{i,j}({\varvec{n}}) \, {\tilde{a}}_{i,j}({\varvec{n}}) \nonumber \\&\quad + s_\textrm{L} \sum _{|{\varvec{n}}-{\varvec{n}}^{\prime }|^2 = 1} \, \sum _{i,j=0,1} {\tilde{a}}^{\dagger }_{i,j}({\varvec{n}}^{\prime }) \, {\tilde{a}}_{i,j}({\varvec{n}}^{\prime }) \,,\end{aligned}$$

10b $$\begin{aligned} {\tilde{\rho }}_{I}({\varvec{n}})&= \sum _{i,j,j^{\prime }=0,1} {\tilde{a}}^{\dagger }_{i,j}({\varvec{n}}) \,\left[ {\varvec{ {\varvec{\tau }}}}_{I}\right] _{j,j^{\prime }} \, {\tilde{a}}_{i,j^{\prime }}({\varvec{n}}) \nonumber \\&\quad + s_\textrm{L} \sum _{|{\varvec{n}}-{\varvec{n}}^{\prime }|^2 = 1} \, \sum _{i,j,j^{\prime }=0,1} {\tilde{a}}^{\dagger }_{i,j}({\varvec{n}}^{\prime }) \,\left[ {\varvec{ {\varvec{\tau }}}}_{I}\right] _{j,j^{\prime }} \, {\tilde{a}}_{i,j^{\prime }}({\varvec{n}}^{\prime }) \,,\end{aligned}$$

10c $$\begin{aligned} {\tilde{\xi }}({\varvec{n}})&= \sum _{i=0,1} {\tilde{b}}^{\dagger }_{i}({\varvec{n}}) \, {\tilde{b}}_{i}({\varvec{n}}) + s_\textrm{L} \sum _{|{\varvec{n}}-{\varvec{n}}^{\prime }|^2 = 1} \, \sum _{i=0,1} {\tilde{b}}^{\dagger }_{i}({\varvec{n}}^{\prime }) \, {\tilde{b}}_{i}({\varvec{n}}^{\prime }) \,. \end{aligned}$$

We also note that, for our lattice MC simulations, we employ the AFQMC approach, which significantly suppresses sign oscillations. The general framework of the AFQMC method for nucleons is described in detail in Ref. [63]. We extend this framework to include hyperons following the approach of Ref. [68]. It is important to note that our calculations consider only $\varLambda$ hyperons, as they are the most significant hyperons known to form bound states in the $S=-1$ sector. Although the $\varLambda$ hyperon is known to mix with the $\varSigma ^0$, we neglect this effect in the current work but will discuss its inclusion later on.

### Hyperon–nucleon interactions

In this section we discuss the details of how to constrain the low-energy constants (LECs) of the *YN* interactions and their incorporation into many-body lattice calculations.

**Fig. 2:**
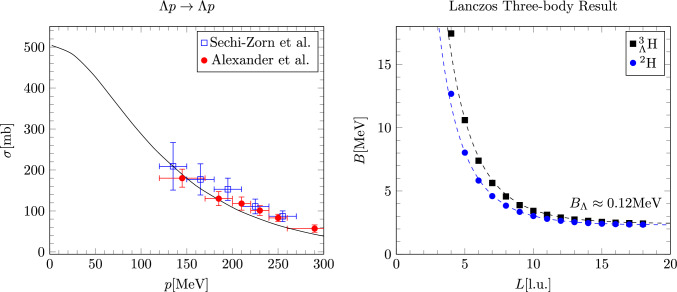
Left panel: Fitted $\varLambda p\rightarrow \varLambda p$ cross section [78, 79]. The coupling constants are chosen to be close to the low-energy scattering parameters of the best continuum chiral SMS N$^2$LO interaction [74], small deviations at higher momenta are therefore expected at leading order. Right panel: Results of an exact Lanczos three-body calculation to confirm that the hypertriton is indeed a shallow bound state for the chosen hypernuclear interaction. The dashed lines are large *L* extrapolations according to Eq. (11)

The LECs of *YN* interactions are determined by fitting the unpolarized cross section at infinite volume limit, while ensuring consistency with the scattering parameters of the best continuum interaction as described in Ref. [74]. Since the splitting of the two S-wave channels, $^1S_0$ and $^3S_1$, cannot be obtained by fitting the cross-section alone, we also use the bound three-body system, the hypertriton, and ensure it maintains a shallow bound state. It is important to note that to accurately calculate the shallow bound state of the hypertriton, we use an exact Lanczos code to eliminate stochastical errors.

Due to the large uncertainty in the empirical $\varLambda$ separation energy for the hypertriton, the corresponding LECs cannot be precisely determined. Nevertheless, the hypertriton serves as a constraint. We note that small variations in the $\varLambda$ separation energy of the hypertriton still allow for an accurate description of heavier hypernuclei [75]. The results for the $\varLambda p \rightarrow \varLambda p$ cross section and the energies of $^2$H and $^3_{\varLambda }$H at finite volumes are shown in Fig. 2.

The extracted scattering parameters from the effective range expansion are close to those obtained with semi-local momentum-space regularized (SMS) N$^2$LO interaction introduced in Ref. [74]. The scattering parameters obtained in this work are summarized in Table 1 and compared with the results of Ref. [74]. For further details on the interaction, see Appendix A.

**Table 1:** Results of scattering lengths and effective ranges for singlet and triplet S-wave $\varLambda N$ scattering phase shifts and comparison with results from the SMS N$^2$LO interaction in $\chi$EFT [74]

	SMS N$^2$LO [74]			This work
	$$\varLambda _{\chi }$$ (MeV)			
	500	550	600	
$a_s$ [fm]	$-$ 2.80	$-$ 2.79	$-$ 2.80	$-$ 2.89
$r_s$ [fm]	2.82	2.89	2.68	3.28
$a_t$ [fm]	$-$ 1.56	$-$ 1.58	$-$ 1.56	$-$ 1.60
$r_t$ [fm]	3.16	3.09	3.17	3.94

Since the hypertriton is very shallow, the $\varLambda -d$ separation is expected to be approximately 11 fm [39]. Therefore, typical box sizes used in lattice calculations with high-fidelity chiral interactions are not sufficient to eliminate all finite volume effects. To address this issue we use a practical solution involving a simplified *NN* interaction^1^ that enables us to use very large boxes, up to $L=24$ fm in our exact calculations. Additionally, we extrapolate to the infinite volume limit using the ansatz [76],

11 $$\begin{aligned} E(L)=E_\infty +\frac{A}{L} \, \exp (-L/L_0) \,, \end{aligned}$$

for both cases, the deuteron and the hypertriton. We expect this two-body formula to be valid according to Ref. [77], since the hypertriton can be subdivided into two-bound clusters, the deuteron and the $\varLambda$.

In our lattice simulations, we follow Ref. [68] and use the AFQMC formulation in a similar manner to include hyperons. As it is discussed in the Supplementary Sections of Ref. [64], in the non-perturbative part of the lattice simulations for nucleons a simple Hamiltonian, which consists of approximate SU(4) symmetric interaction, is used and it is defined as,

12 $$\begin{aligned} \begin{aligned} H^S&= H^{N}_\textrm{free} + \frac{C_{NN}}{2}\sum _{{\varvec{n}}}: \left[ {\tilde{\rho }}({\varvec{n}}) \right] ^2 : \\&\quad + \frac{C_{NN}^{I}}{2}\sum _{I,{\varvec{n}}}: \left[ {\tilde{\rho }}_{I}({\varvec{n}}) \right] ^2 : + V_\textrm{OPE} , \end{aligned} \end{aligned}$$

where $H^{N}_\textrm{free}$ is the kinetic energy term for nucleons defined by using fast Fourier transforms to produce the exact dispersion relations $E_{N} =p^2/(2m_{N})$ with nucleon mass $m_{N}=938.92$ MeV, $C_{NN}$ is the coupling constant of the short-range SU(4) symmetric interaction, $C_{NN}^{I}$ is the coupling constant of the short-range isospin breaking interaction, $V_\textrm{OPE}$ is the long-range one-pion-exchange (OPE) potential for nucleons, the  :  :  symbol indicates normal ordering. We use local smearing parameter $s_\textrm{L}=0.07$ and non-local smearing parameter $s_\textrm{NL} = 0.5$ as they are set in Ref. [64]. To consider non-perturbative contributions for the *YN* interactions, we define a spin-averaged *YN* interaction,

13 $$\begin{aligned} C_{Y N} = \frac{1}{4}(C_{YN}^S + 3\,C_{YN}^T)\,, \end{aligned}$$

which enables us to derive an auxiliary field formulation for systems consisting of nucleons and $\varLambda$ hyperons. To end this, we redefine the second term of Eq. (12) as,

14 $$\begin{aligned} \frac{C_{NN}}{2}\sum _{{\varvec{n}}}: \left[ {\tilde{\rho }}({\varvec{n}}) \right] ^2 : + C_{Y N} \sum _{{\varvec{n}}} : {\tilde{\rho }}({\varvec{n}}) {\tilde{\xi }}({\varvec{n}}) :\,. \end{aligned}$$

The expression given in Eq. (14) can rewritten by completing the square as15We note that this expression induces a *YY* interaction, which, however, due to the absence of a second $\varLambda$ is zero by construction. While this modified density allows parallel treatment of nuclear as well as hypernuclear contact interactions with one auxiliary field, we observe degeneracy for the states with different total spin states in hypernuclei due to the spin-averaged *SU*(4) symmetric interaction. Nevertheless, the corrections to the spin-averaged *SU*(4) symmetric interaction,  are treated using first order perturbation theory, and for systems with a low-lying excited state we lift the degeneracy of ground and excited states using degenerate perturbation theory based on the nuclear ground state, see also Appendix B. Those separation of states within e.g. $A=4$ nucleus are of particular interest since they are measured very accurately and are sensitive to the spin of the $\varLambda$.

### Fit of the three-body forces

In this section, we discuss the details of the three-baryon interactions $V_{YNN}$ given in Eq. (2) and how we regulate their singular short-distance properties. Recent *ab-initio* calculations have shown that the locality of the short range interactions plays significant role on determination of the properties of atomic nuclei [64, 80]. Therefore, we construct and analyse the interactions given in Eq. (2) with all possible choice of smearing, both locally and non-locally. Such a procedure is not only important to get an accurate description for the properties of hypernuclei but also it is important to emulate the range of missing meson exchange forces.

Hence we introduce the locally smeared forms of the interactions given in Eq. (2),

16 $$\begin{aligned} \begin{aligned} V_{1}^{(d)}&= 3 \, \sum _{{\varvec{n}}} : \left\{ [\rho ^{(d)}({\varvec{n}})]^2 \, - \sum _{S} \, [\rho _{S}^{(d)}({\varvec{n}})]^2 \right\} \xi ^{(d)}({\varvec{n}}): \\&\quad + \sum _{{\varvec{n}},I} : \left\{ [\rho _{I}^{(d)}({\varvec{n}})]^2 - \sum _{S} \, [\rho _{SI}^{(d)}({\varvec{n}})]^2 \right\} \xi ^{(d)}({\varvec{n}}) : \,, \end{aligned} \end{aligned}$$

17 $$\begin{aligned} \begin{aligned} V_{2}^{(d)}&= 2 \, \sum _{{\varvec{n}}}: \rho ^{(d)}({\varvec{n}}) \sum _{S} \rho _{S}^{(d)}({\varvec{n}}) \xi _{S}^{(d)}({\varvec{n}}) :\qquad \qquad \qquad \quad \\&\quad -2 \, \sum _{{\varvec{n}},S,I}: \rho _{I}^{(d)}({\varvec{n}}) \rho _{SI}^{(d)}({\varvec{n}}) \xi _{S}^{(d)}({\varvec{n}}): \,, \end{aligned} \end{aligned}$$

18 $$\begin{aligned} \begin{aligned} V_{3}^{(d)}&= 3 \, \sum _{{\varvec{n}}} : \left\{ [\rho ^{(d)}({\varvec{n}})]^2 \, - \sum _{I} \, [\rho _{I}^{(d)}({\varvec{n}})]^2 \right\} \xi ^{(d)}({\varvec{n}}) : \\&\quad + \sum _{{\varvec{n}},S} : \left\{ [\rho _{S}^{(d)}({\varvec{n}})]^2 - \sum _{I} \, [\rho _{SI}^{(d)}({\varvec{n}})]^2 \right\} \xi ^{(d)}({\varvec{n}}) : \,. \end{aligned} \end{aligned}$$

Here, the superscript *d* describes the range of the local smearing, and we consider different choices up to $d = 3$ which corresponds to 2.28 fm. In addition, for these interactions we set $s_\textrm{L} = 0.5$. Therefore, the locally smeared three-body interactions are labelled as $V_{k}^{(d=0)}$, $V_{k}^{(d=1)}$, $V_{k}^{(d=2)}$ and $V_{k}^{(d=3)}$ with $k = 1,2,3$. We also define the non-locally smeared forms of the interactions given in Eq. (2),

19 $$\begin{aligned} \begin{aligned} V_{1}^{s_\textrm{NL}}&= 3 \, \sum _{{\varvec{n}}} : \left\{ [{\hat{\rho }}({\varvec{n}})]^2 \, - \sum _{S} \, [{\hat{\rho }}_{S}({\varvec{n}})]^2 \right\} {\hat{\xi }}({\varvec{n}}) : \\&\quad + \sum _{{\varvec{n}},I} : \left\{ [{\hat{\rho }}_{I}({\varvec{n}})]^2 - \sum _{S} \, [{\hat{\rho }}_{SI}({\varvec{n}})]^2 \right\} {\hat{\xi }}({\varvec{n}}) : \,, \end{aligned}\end{aligned}$$

20 $$\begin{aligned} \begin{aligned} V_{2}^{s_\textrm{NL}}&= 2 \, \sum _{{\varvec{n}}}: {\hat{\rho }}({\varvec{n}}) \sum _{S} {\hat{\rho }}_{S}({\varvec{n}}) {\hat{\xi }}_{S}({\varvec{n}}): \qquad \qquad \qquad \quad \\&\quad -2 \, \sum _{{\varvec{n}},S,I}: {\hat{\rho }}_{I}({\varvec{n}}) {\hat{\rho }}_{SI}({\varvec{n}}) {\hat{\xi }}_{S}({\varvec{n}}): \,, \end{aligned} \end{aligned}$$

21 $$\begin{aligned} \begin{aligned} V_{3}^{s_\textrm{NL}}&= 3 \, \sum _{{\varvec{n}}} : \left\{ [{\hat{\rho }}({\varvec{n}})]^2 \, - \sum _{I} \, [{\hat{\rho }}_{I}({\varvec{n}})]^2\right\} {\hat{\xi }}({\varvec{n}}) : \\&\quad + \sum _{{\varvec{n}},S} : \left\{ [{\hat{\rho }}_{S}({\varvec{n}})]^2 - \sum _{I} \, [{\hat{\rho }}_{SI}({\varvec{n}})]^2 \right\} {\hat{\xi }}({\varvec{n}}) : \,. \end{aligned} \end{aligned}$$

Here, the superscript ${s_\textrm{NL}}$ indicates the strength of the non-locality of the interactons. We consider three different values of the parameter ${s_\textrm{NL}}=0.1, 0.2, 0.3$, labeled $V_{k}^{{s_\textrm{NL}}=0.1}$, $V_{k}^{{s_\textrm{NL}}=0.2}$ and $V_{k}^{{s_\textrm{NL}}=0.3}$ with $k = 1,2,3$. In the following, we reduce the superscript of these interaction and they are denoted by $V_{k}^{0.1}$, $V_{k}^{0.2}$ and $V_{k}^{0.3}$.

Now we consider all possible versions of the $\varLambda NN$ interaction, constructed from the combinations of these smeared versions of $V_{1}$, $V_{2}$, and $V_{3}$, which leads to a total set of 343 combinations. By systematically analyzing each combination using hypernuclei from light to medium mass, we determine the optimal configuration for the $\varLambda NN$ interaction which give s a good description for hypernuclei. An analysis of such kind revisiting the nuclear three-body forces is in preparation by some of the authors.

In order to increase the predictive power of the theory we want to fit to a limited set of hypernuclei. We distinguish here between two different types of hypernuclei. Those with an $\alpha$-like nuclear core, which are insensitive to details of the spin-dependent force and the ones that exhibit a high sensitivity to spin-dependent forces by having a low-lying excited state. While the latter are needed to extract $C_2$, $\alpha$-like hypernuclei are important to scale correctly towards the medium-mass region and permit to access the overall strength of $C_{1}$ and $C_{3}$.

We do a least square fit to determine the LECs of $\varLambda NN$ interaction. However, due to the lack of explicit two-pion exchange interactions, smeared forces emulate the long-range part of the potential and hence introduce an additional amount of freedom as mentioned before. Therefore, in order to estimate the quality of our description we use the Root Mean Square Deviation (RMSD) defined as follows

22 $$\begin{aligned} \text {RMSD}(S)=\sqrt{\frac{1}{M_S}\sum _{i\in S}\left( \frac{^{i}B^c_\varLambda - ^{i}B^{\exp }_\varLambda }{^{i}B^{\exp }_\varLambda }\right) ^2}, \end{aligned}$$

where $^i B_\varLambda ^c$ is the evaluated $\varLambda$ separation energy and $^i B^{\exp }_\varLambda$ is the experimental separation energy for each hypernucleus within the set. The size of the set of hypernuclei is given by $M_S$ of the set *S*, which contains well measured hypernuclei from the light and medium mass region, starting from $A=4$ up to $A=16$. The corresponding $^i B^{\exp }_\varLambda$ are taken from Ref. [81]. For reference the RMSD for the calculation without hypernuclear three-body forces is $\text {RMSD}_{\text {no }\varLambda \text {NN}}(S)=18.4\%$.

In order to exemplify how well the *YNN* forces can be constrained from light hypernuclei, we consider here two scenarios. In scenario 1, we constrain the three-body forces only by the light $A=4$ and $A=5$ system, while in scenario 2 we use hypernuclei up to $A=16$. Since the splitting between the ground state and the excited state in the hydrogen and the helium four-body system is supposedly a charge symmetry effect [29], which we cannot resolve with the current setup, we take here the average.

A good starting point of choosing such hypernuclear three-body forces is decuplet saturation as described in Ref. [72]. In this approach $C_3=C_1$ and $C_2=0$, since equal coupling strengths require that the forces are equally smeared, only seven combination of three-body forces remain. We start from this assumption and gradually relax the constraints to obtain sets of three-body *YNN* forces that describe the data better and better. We expect that deviations from decuplet saturation are needed in the end since we do not yet include the explicit $\varLambda -\varSigma ^0$ conversions. The seven different combinations are listed in Table 2 with the best interaction pair resulting in an RMSD of $9.3\%$ while the worst has an RMSD of 14.7%.

**Table 2:** Possible combination of *YNN* forces enforcing decuplet saturation $C_3=C_1$ and $C_2=0$, sorted by the RMSD fitted to the four- and five-body system. The most right column gives the result fitted to the complete set of hypernuclei (scenario 2)

Interaction	RMSD (%)	
$V_{YNN}$	$A=4/5$	$A\ge 4$
$V_{1}^{0.1}+V_{3}^{0.1}$	9.3	9.2
$V_{1}^{0.2}+V_{3}^{0.2}$	9.5	9.3
$V_{1}^{0.3}+V_{3}^{0.3}$	9.9	9.7
$V_{1}^{(d = 0)}+V_{3}^{(d = 0)}$	12.9	12.6
$V_{1}^{(d = 1)}+V_{3}^{(d = 1)}$	14.7	14.6
$V_{1}^{(d = 2)}+V_{3}^{(d = 2)}$	14.7	14.6
$V_{1}^{(d = 3)}+V_{3}^{(d = 3)}$	14.7	14.6

In the next step we try to improve the description by allowing the $\varLambda$ spin-dependent force, parameterized by $C_2$, to be non-zero. In total 21 of the 49 combination of forces improve the overall result. In order to keep the main text readable we only list the five combinations with the least RMSD in Table 3. For the full list we refer to Appendix C. Since this three-body force depends on the spin of the $\varLambda$, it can directly influence the splitting of the four-body system, and therefore we see a significant improvement of the RMSD. The best interaction set has an RMSD of $5.7\%$, while the worst that still improves the interaction has an RMSD of $9.1\%$. In addition, it confirms that our original set of non-local three-body forces is sufficient, since the weaker smeared force produces a better result. Thus non-local smearing parameters larger than ${s_\textrm{NL}}=0.3$ are unlikely to provide a more accurate description.

**Table 3:** The 5 combinations with the least RMSD when constrained via the four- and five-body systems with $C_3=C_1$. A complete list can be found in Appendix C

Interaction	RMSD (%)	
$V_{YNN}$	$A=4/5$	$A\ge 4$
$V^{0.1}_1+V^{(d = 0)}_2+V^{0.1}_3$	5.7	5.3
$V^{0.2}_1+V^{(d = 0)}_2+V^{0.2}_3$	5.8	5.4
$V^{0.3}_1+V^{(d = 0)}_2+V^{0.3}_3$	6.3	5.9
$V^{0.1}_1+V^{(d = 3)}_2+V^{0.1}_3$	7.6	7.4
$V^{0.2}_1+V^{(d = 3)}_2+V^{0.2}_3$	7.7	7.5

In a last step we take all 343 combinations into account and find 27 combinations that improve the description when fitting from the $A=4$ and $A=5$ hypernuclei.^2^ Interestingly none of those features a smeared $\varLambda$ spin-dependent force. The best interactions show here an RMSD of $3.7\%$ for the first scenario, while the less effective are as good as the best restricted ones. We list again the five combinations with the least RMSD in Table 4.

**Table 4:** The 5 combinations with the least RMSD when constrained via the four- and five-body systems. A complete list can be found in Appendix C

Interaction	RMSD (%)	
$V_{YNN}$	$A=4/5$	$A\ge 4$
$V^{(d=2)}_1+V^{(d=0)}_2+V^{(d=1)}_3$	3.7	3.6
$V^{(d=1)}_1+V^{(d=0)}_2+V^{(d=2)}_3$	3.7	3.7
$V^{(d=1)}_1+V^{(d=0)}_2+V^{(d=1)}_3$	3.8	3.7
$V^{(d=1)}_1+V^{(d=0)}_2+V^{(d=3)}_3$	3.9	3.7
$V^{(d=1)}_1+V^{(d=0)}_2+V^{(d=0)}_3$	4.1	4.1

For this procedure we use well measured hypernuclei in the low-mass region. For a comprehensive overview of hypernuclear binding energies, see for example [81]. We also want to give a short discussion on the development of the coupling constants for the corresponding best interactions for scenario one. In case of decuplet saturation the coupling constant $C_1=C_3$ is given by $C_1=-0.00105$. Turning on the coupling constant for the second three-body force, the coupling constant $C_1$ changes to $C_1=-0.00145$, while $C_2=-0.22051$. We want to point out that the strength of these coupling constants should not be compared directly to each other, since $C_2$ does not feature smearing. This also makes the comparison to the full result difficult, since the structure (smearing) changes completely. We obtain $C_1=-0.01608$, $C_2=-0.27524$ and $C_3=0.01596$. We notice some cancellations between $C_1$ and $C_3$, which we naively expect to be necessary at LO, however, one should not directly compare those numbers to the ones obtained before, due to different smearings. For further details on the fitting procedure see also Appendix C.

### Finite volume effects

In addition to the previously discussed hypertriton, which exhibits significant sensitivity to the box size due to its extended structure (for details on the MC simulations extrapolation, see Appendix D), we briefly examine the dependence on the box size *L* for another critical set of hypernuclei, namely the four-body system.

As the separation energies increase, we anticipate that box sizes typically used in nuclear calculations will suffice, since the $\varLambda$ binding energies begin to exceed typical nuclear binding energies per nucleon. This is already evident in the four-body system.

**Fig. 3:**
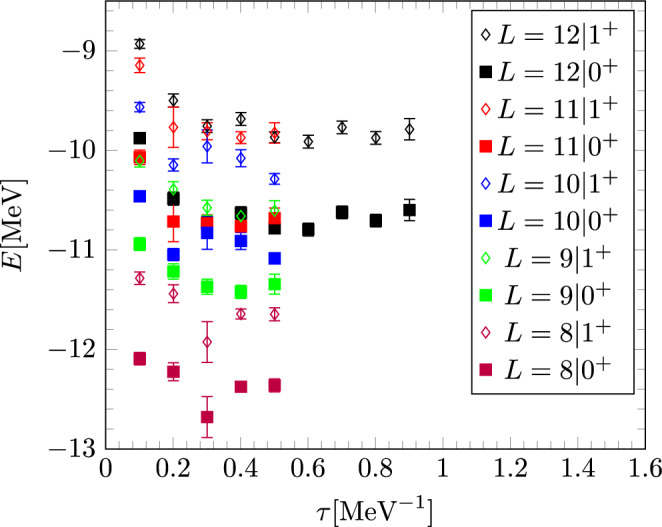
N$^3$LO energies for different box sizes (in units of *a*) for the excited (diamonds) as well as the ground state (squares) of $^4_\varLambda$H on the two-body level. The black points are used for the euclidean time extrapolation for the final result

In Fig. 3, we present the behavior of the $0^+$ and $1^+$ states in $^4_\varLambda$H at the two-body level. The differences between the smaller box sizes and those used for extrapolation with $L=12$ are minimal. Thus, finite box size effects are well-controlled for hypernuclei starting from the four-body system.

## Results and discussion

We calculate the ground state and excited state energies of hypernuclei up to $$A = 16$$. Our calculations employ high-fidelity chiral interactions at N$$ ^3$$LO for nucleons, as developed in [64], leading-order S-wave hyperon-nucleon (*YN*) interactions constrained by the unpolarized $$\varLambda p \rightarrow \varLambda p$$ cross section and the hypertriton binding energy, and hyperon-nucleon-nucleon (*YNN*) interactions constrained by hypernuclear systems with $$A = 4$$ and 5. For the *YNN* interactions, we consider all possible forms of short-distance smearing. In our analysis, we calculate the RMSD over all calculated hypernuclear separation energies with $$A\ge 4$$, which are used to assess the accuracy of the *YNN* interactions in describing hypernuclei.

**Fig. 4:**
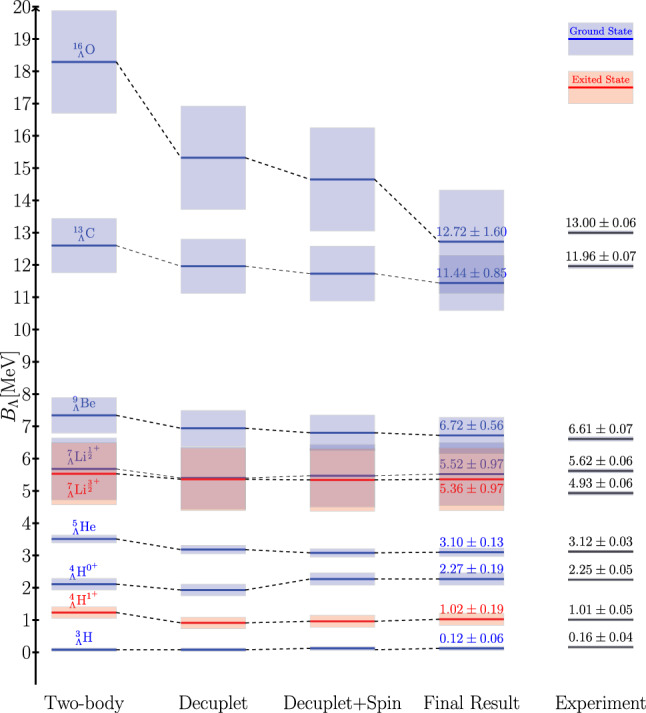
$\varLambda$ separation energies for different *YNN* forces in scenario 1. We choose the best combination for each proposed set. The large improvement resulting from the introduction of the spin-dependent three-body force is clearly visible. The experimental values are taken from [81], where we averaged the four-body systems. Ground states are depicted in blue, excited states in red. The uncertainties are indicated by the shaded areas

We present results based on only two-body *YN* interactions, *YN* interactions combined with the best set of *YNN* interactions with decuplet approximations, and *YN* and *YNN* interactions with fitted LECs in Fig. 4 and in Table 5. The results for $A \le 5$ shown here are included in the fit, while the other hypernuclei are predictions. We find that, within stochastic uncertainties of the Monte Carlo simulations, our Hamiltonian can accurately describe hypernuclear systems.

**Table 5:** $\varLambda$ separation energies (in MeV) for different *YNN* forces in scenario 1. We choose the best combination (least RMSD) for each proposed set. The large improvement resulting from the introduction of the spin-dependent three-body force is clearly visible. The experimental values are taken from [81], where we averaged the four-body systems

Nucleus	Exp.	Two-body (YN)	$V_{\text {Decuplet}}$	$V_{\text {Decuplet}}\,+\,V_2\,$	Free $V_1\,+\,V_2\,+\,V_3\,$
$^3_\varLambda \text {H}$	$0.16\pm {0.04}$	$0.08\pm {0.05}$	$0.08\pm {0.05}$	$0.12\pm {0.06}$	$0.12\pm {0.06}$
$^4_\varLambda \text {H}^{0^+}$	$2.25\pm {0.042}$	$2.11\pm {0.18}$	$1.93\pm {0.18}$	$2.27\pm {0.19}$	$2.258\pm {0.19}$
$^4_\varLambda \text {H}^{1^+}$	$1.01\pm {0.046}$	$1.23\pm {0.18}$	$0.95\pm {0.18}$	$0.97\pm {0.19}$	$1.012\pm {0.19}$
$^5_\varLambda \text {He}$	$3.102\pm {0.03}$	$3.51\pm {0.12}$	$3.22\pm {0.12}$	$3.11\pm {0.12}$	$3.10\pm {0.13}$
$^7_\varLambda \text {Li}^{\frac{1}{2}^+}$	$5.62\pm {0.06}$	$5.68\pm {0.96}$	$5.40\pm {0.97}$	$5.47\pm {0.97}$	$5.52\pm {0.97}$
$^7_\varLambda \text {Li}^{\frac{3}{2}^+}$	$4.93\pm {0.06}$	$5.53\pm {0.96}$	$5.36\pm {0.97}$	$5.34\pm {0.97}$	$5.36\pm {0.97}$
$^9_\varLambda \text {Be}$	$6.61\pm {0.07}$	$7.34\pm {0.55}$	$6.94\pm {0.56}$	$6.80\pm {0.55}$	$6.72\pm {0.55}$
$^{13}_{\varLambda }\text {C}$	$11.96\pm {0.07}$	$12.60\pm {0.84}$	$11.96\pm {0.45}$	$11.73\pm {0.85}$	$11.44\pm {0.84}$
$^{16}_{\varLambda }\text {O}$	$13.00\pm {0.06}$	$18.29\pm {1.59}$	$15.32\pm {1.60}$	$14.65\pm {1.60}$	$12.72\pm {1.61}$

To analyze any further improvements in the results given in Table 5, we also perform fits of *YNN* interactions and RMSD analysis by considering hypernuclei up to $A=16$. The results are shown in Table 6, and we find that the improvement in the final results is minimal, as the data is already well-described within uncertainties. However, the inclusion of heavier hypernuclei may be necessary in the future to address mid- to heavy-mass hypernuclei.

**Table 6:** $\varLambda$ separation energies (in MeV) for different *YNN* forces in scenario 2. We choose the best combination (least RMSD) for each proposed set. The large improvement resulting from the introduction of the spin-dependent three-body force is clearly visible. The experimental values are taken from [81], where we averaged the four-body systems

Nucleus	Exp.	Two-body (YN)	$V_{\text {Decuplet}}$	$V_{\text {Decuplet}}\,+\,V_2\,$	Free $V_1\,+\,V_2\,+\,V_3\,$
$^3_\varLambda \text {H}$	$0.16\pm {0.04}$	$0.08\pm {0.05}$	$0.08\pm {0.05}$	$0.12\pm {0.06}$	$0.12\pm {0.06}$
$^4_\varLambda \text {H}^{0^+}$	$2.25\pm {0.042}$	$2.11\pm {0.18}$	${1.90}\pm {0.18}$	$2.27\pm {0.19}$	$2.252\pm {0.19}$
$^4_\varLambda \text {H}^{1^+}$	$1.01\pm {0.046}$	$1.23\pm {0.18}$	$0.91\pm {0.18}$	$0.96\pm {0.19}$	$1.023\pm {0.19}$
$^5_\varLambda \text {He}$	$3.102\pm {0.03}$	$3.51\pm {0.12}$	$3.18\pm {0.13}$	$3.08\pm {0.13}$	$3.11\pm {0.13}$
$^7_\varLambda \text {Li}^{\frac{1}{2}^+}$	$5.62\pm {0.06}$	$5.68\pm {0.96}$	$5.35\pm {0.97}$	$5.45\pm {0.97}$	$5.51\pm {0.97}$
$^7_\varLambda \text {Li}^{\frac{3}{2}^+}$	$4.93\pm {0.06}$	$5.53\pm {0.96}$	$5.32\pm {0.97}$	$5.32\pm {0.97}$	$5.37\pm {0.97}$
$^9_\varLambda \text {Be}$	$6.61\pm {0.07}$	$7.34\pm {0.55}$	$6.89\pm {0.55}$	$6.76\pm {0.55}$	$6.73\pm {0.56}$
$^{13}_{\varLambda }\text {C}$	$11.96\pm {0.07}$	$12.60\pm {0.84}$	$11.87\pm {0.84}$	$11.66\pm {0.85}$	$11.47\pm {0.85}$
$^{16}_{\varLambda }\text {O}$	$13.00\pm {0.06}$	$18.29\pm {1.59}$	$14.86\pm {1.60}$	$14.36\pm {1.60}$	$13.00\pm {1.61}$

As seen from Fig. 4 and Tables 5 and  6, the contributions from three-body forces exhibit the expected behavior. The splitting in the $A=4$ sector can be described accordingly by introducing a spin-dependent *YNN* force. We also observe that the uncertainties in the MC simulations are dominated by operators that are only sensitive to the nuclear part of the wave function rather than the operators of the *YN* interaction. Additionally, the uncertainties in atomic nuclei, as described in Ref. [64], currently represent lower limits on accuracy of the separation energy. For the separation between the ground and excited states, the splitting can be determined more precisely than the results suggest due to correlated uncertainties. For further details, see also Appendix E.

For the hypertriton, we need to extrapolate to larger box sizes, and information on the extrapolation procedure can be found in Appendix D. Despite using a simplified nuclear interaction for the fitting procedure, our results are fully compatible with experimental measurements as well as our original fit to determine the coupling constants. The contribution from three-body interactions is about 40 keV.

Compared to the splitting in the $A=4$ sector, the splitting in the $A=7$ sector, although within uncertainties, is relatively inaccurate. We anticipate immediate improvement by introducing meson exchanges, particularly pions. This is because $\ell =2$ contributions from the nuclear core, which are missing in the degenerate perturbation theory construction based on the $1^+$ ground state, can contribute to the $\frac{3}{2}^+$ state (see Appendix B). These contributions will be then automatically included.

This leads us to possible improvements in the considered interactions here. We recommend including pion exchange forces in both the two-body and three-body sector. These forces not only allow for an automatic inclusion of higher momentum contributions but also make excited states available in typical multichannel calculations, as used for example in [64, 82, 83], instead of relying on perturbation theory. Since the main contribution at the two-body level comes from two-pion exchange interactions rather than one-pion exchange interaction, we expect the sign oscillation to be under control. Additionally, this approach enables the inclusion of higher orders in the chiral scheme, necessary for better phase shift descriptions at higher orders, including higher partial waves such as P-waves in the $\varLambda N$ sector, which are important for describing higher excited states in light hypernuclei like $^7_\varLambda$Li and $^9_\varLambda$Be.

In the final step, the explicit inclusion of the $\varSigma$ and the $\varLambda - \varSigma ^0$ conversion could further improve the results. This inclusion is feasible in a manner similar to the $\varLambda$ inclusion in this work and Ref. [68], or using a perturbative scheme as suggested in Ref. [84]. Such conversions are crucial for describing light hypernuclei within the NCSM framework [28–34].

In this work we extend the successful N$^3$LO nuclear interaction towards light and medium mass hypernuclei based on a LO  $\varLambda N$ interaction. We present results describing accurately the ground state as well as exited states of selected hypernuclei by including contact three-body *YNN* forces constrained only from the light $A=4,5$ system. This is an important step towards the *ab initio* description of hypernuclei in the framework of NLEFT.

## Figures and Tables

**Fig. 5:**
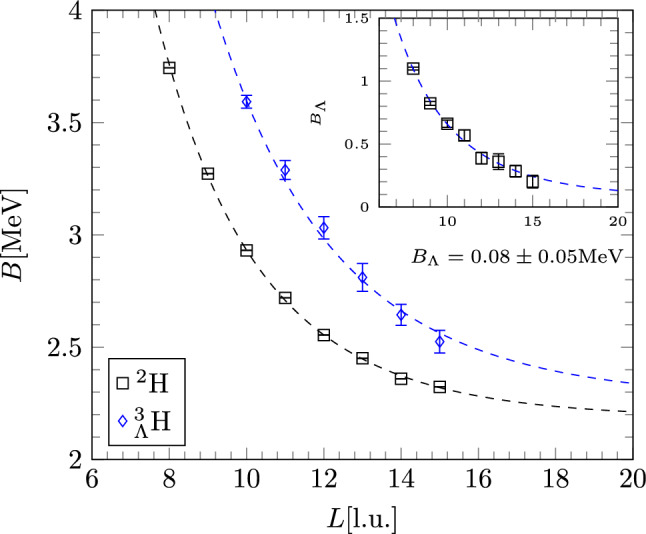
Infinite volume extrapolation of the ground state energies of the deuteron and the hypertriton from high-fidelity interactions. The inset shows the extrapolation of the separation energy

**Fig. 6:**
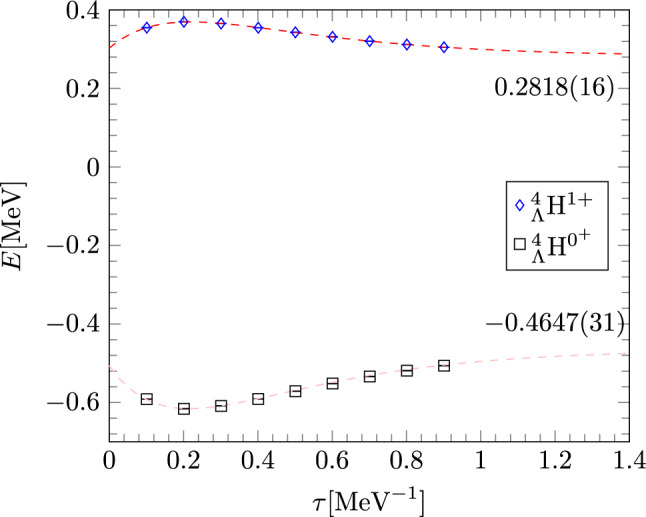
Contribution of the spin-dependent part of the YN interaction to the two four-body energy levels in DPT on a two-body level if extracted independently from the nuclear part. The dashed lines indicate the Euclidean time extrapolations, the difference of those contributions results in the splitting between the ground and excited state

## Data Availability

Data will be made available on reasonable request. [Author’s comment: The datasets generated during and/or analysed during the current study are available from the corresponding author on reasonable request.]

## Interaction details

In this work we use a spatial lattice spacing of $a=1.32$ fm and a temporal lattice spacing of $a_t=1/1000\,$MeV$^{-1}$. All interactions share a similar set of local and non-local smearing parameters of $s_\textrm{L}=0.07$ and $s_\textrm{NL}=0.5$, the same parameters are also used in Ref. [64] for the *NN* interaction. The presence of non-local smearing results in an explicit dependence on the center-of-mass momentum, thus breaking Galilean invariance. As it is considered for nucleon-nucleon interactions in Ref. [64], in this work we include the nucleon-hyperon Galilean Invariance Restoration (GIR) interaction. For an extensive introduction to GIR interactions on the lattice, see Ref. [85]. The LECs of the *YN* interactions are given in Table 7.

**Table 7:** Coupling constants of *YN* interactions as well as the corresponding GIR interactions (in lattice units)

	$^1S_0(YN)$	$^3S_1(YN)$
*C*	$-3.16\times 10^{-3}$	$-2.39\times 10^{-3}$
$C_\text {GIR}^0$	$-4.36\times 10^{-4}$	$-3.28\times 10^{-4}$
$C_\text {GIR}^1$	$8.95\times 10^{-5}$	$6.73\times 10^{-5}$
$C_\text {GIR}^2$	$-8.40\times 10^{-6}$	$-6.31\times 10^{-6}$

In the evolution in our MC simulation we than use the spin averaged force $C_{\text {YN}}=-2.58\times 10^{-3}$.

## Construction based on degenerate perturbation theory

As discussed in Sect. 2.1, we construct a unified *ab initio* theory to study hypernuclei building upon the *ab initio* theory for nuclei developed in Ref. [64]. By incorporating hyperons into the theory for nucleons, we employ a recently formulated AFQMC method [68], which considers only spin and isospin symmetric *YN* interaction in the non-perturbative calculations. Such a choice enables efficient calculations for the systems consisting of nucleons and hyperons, but at the cost of observing degeneracy in the low-lying energy levels for some hypernuclei, such as $^{4}_{\varLambda }$H and $^{7}_{\varLambda }$Li. To lift such type of degeneracy we treat the spin breaking *YN* and *YNN* interactions using degenerate perturbation theory (DPT), an essential technique for accurately describing the behavior of systems with overlapping energy levels. In DPT, the process involves constructing the perturbation Hamiltonian within the degenerate subspace and then diagonalizing it to find the new energy eigenvalues and corresponding eigenstates.

For instance, we consider the $^{4}_{\varLambda }$H hypernucleus where we start with the nuclear ground state (e.g., the triton, which is a $J=1/2$ state due to the Pauli exclusion principle between nucleons) and add an additional $\varLambda$. To address the two lowest-lying energy states, we perform a multi-state calculation with quantum numbers $J=0, J_z=0$ and $J=1, J_z=0$ using the following initial wave functions for the $^{4}_{\varLambda }$H system,

23a $$\begin{aligned} \vert {\psi _1} \rangle&=a^{\dagger }_{\uparrow ,p}(0)a^{\dagger }_{\uparrow ,n}(0)a^{\dagger }_{\downarrow ,n}(0)b^{\dagger }_{\downarrow }(0)\vert {0} \rangle ~, \end{aligned}$$

23b $$\begin{aligned} \vert {\psi _2} \rangle&=a^{\dagger }_{\downarrow ,p}(0)a^{\dagger }_{\downarrow ,n}(0)a^{\dagger }_{\uparrow ,n}(0)b^{\dagger }_{\uparrow }(0)\vert {0} \rangle ~, \end{aligned}$$

where the creation operators the ones defined in the main text.

**Table 8:** Combination that improve the least RMSD when constrained via the four- and five-body systems with $C_3=C_1$ compared to the best fit considering decuplet saturation

Interaction	RMSD (%)	
$V_{YNN}$	$A=4/5$	$A\ge 4$
$V^{0.1}_1+V^{(d=0)}_2+V^{0.1}_3$	5.7	5.3
$V^{0.1}_1+V^{(d=1)}_2+V^{0.1}_3$	7.8	7.6
$V^{0.1}_1+V^{(d=2)}_2+V^{0.1}_3$	7.7	7.5
$V^{0.1}_1+V^{(d=3)}_2+V^{0.1}_3$	7.6	7. 4
$V^{0.1}_1+V^{0.1}_2+V^{0.1}_3$	8.7	8.1
$V^{0.1}_1+V^{0.2}_2+V^{0.1}_3$	8.7	8.1
$V^{0.1}_1+V^{0.3}_2+V^{0.1}_3$	8.7	8.1
$V^{0.2}_1+V^{(d=0)}_2+V^{0.2}_3$	5.8	5.4
$V^{0.2}_1+V^{(d=1)}_2+V^{0.2}_3$	7.9	7.6
$V^{0.2}_1+V^{(d=2)}_2+V^{0.2}_3$	7.8	7.6
$V^{0.2}_1+V^{(d=3)}_2+V^{0.2}_3$	7.7	7.5
$V^{0.2}_1+V^{0.1}_2+V^{0.2}_3$	8.8	8.2
$V^{0.2}_1+V^{0.2}_2+V^{0.2}_3$	8.8	8.2
$V^{0.2}_1+V^{0.3}_2+V^{0.2}_3$	8.8	8.2
$V^{0.3}_1+V^{(d=0)}_2+V^{0.3}_3$	6.3	5.9
$V^{0.3}_1+V^{(d=1)}_2+V^{0.3}_3$	8.3	8.0
$V^{0.3}_1+V^{(d=2)}_2+V^{0.3}_3$	8.2	7.9
$V^{0.3}_1+V^{(d=3)}_2+V^{0.3}_3$	8.1	7.9
$V^{0.3}_1+V^{0.1}_2+V^{0.3}_3$	9.1	8.5
$V^{0.3}_1+V^{0.2}_2+V^{0.3}_3$	9.1	8.5
$V^{0.3}_1+V^{0.3}_2+V^{0.3}_3$	9.1	8.5

We then compute the corresponding $2\times 2$ perturbation Hamiltonian for any operator ${\mathcal {O}}$ involving hyperons,

24 $$\begin{aligned} H_{{\mathcal {O}}}^{\tau } = \begin{pmatrix} \langle {\psi _1^{\tau }}|{{\mathcal {O}}}|{\psi _1^{\tau }}\rangle & \quad \langle {\psi _1^{\tau }}|{{\mathcal {O}}}|{\psi _2^{\tau }}\rangle \\ \langle {\psi _2^{\tau }}|{{\mathcal {O}}}|{\psi _1^{\tau }}\rangle & \quad \langle {\psi _2^{\tau }}|{{\mathcal {O}}}|{\psi _2^{\tau }}\rangle \end{pmatrix}\,, \end{aligned}$$

where $\psi _{1,2}^{\tau }$ is the projected state at a given Euclidean time $\tau$. Due to the absence of any meson-exchange interaction between nucleons and hyperons, the relations between the entries of the perturbation Hamiltonian given in Eq. (24), up to a stochastic error, can be written as $\langle {\psi _1^{\tau }}|{{\mathcal {O}}}|{\psi _1^{\tau }}\rangle \approx \langle {\psi _2^{\tau }}|{{\mathcal {O}}}|{\psi _2^{\tau }}\rangle$ and $\langle {\psi _1^{\tau }}|{{\mathcal {O}}}|{\psi _2^{\tau }}\rangle \approx \langle {\psi _2^{\tau }}|{{\mathcal {O}}}|{\psi _1^{\tau }}\rangle$. As a result, the diagonalization of Eq. (24) yields the eigenenergies for $^{4}_{\varLambda }$H$^{1^+}$ and $^{4}_{\varLambda }$H$^{0^+}$ with the corresponding eigenstates

25a

25b

We apply a similar procedure for the $A=7$-body system, although the nuclear core in this case is an $\ell =1$ state. Since this method is based solely on the nuclear ground state, contributions from excited nuclear states (which can particularly affect excited states in the $A=7$ system) are missing. This limits our predictive power for such systems. In future studies, a full inclusion of meson-exchange will remove the dependence on this type of perturbative calculation for excited states.

## Fitting procedure

In this section, we present a comprehensive analysis of the *YNN* interactions by considering all possible combinations of the interactions out of $\lbrace V_{1}^{(d)}, V_{1}^{s_\textrm{NL}}\rbrace$, $\lbrace V_{2}^{(d^{\prime })}, V_{2}^{s_\textrm{NL}^{\prime }}\rbrace$ and $\lbrace V_{3}^{(d^{\prime \prime })}, V_{3}^{s_\textrm{NL}^{\prime \prime }}\rbrace$. Such a detailed analysis is feasible because the *YNN* interactions are treated perturbatively in our calculations.

**Table 9:** Combinations of YNN-forces constrained via the four- and five-body systems that reduce the RMSD when compared with the best combination given in Table 3, by lifting all constraints

Interaction	RMSD (%)	
$V_{YNN}$	$A=4/5$	$A\ge 4$
$V^{(d=0)}_1+V^{(d=0)}_2+V^{(d=1)}_3$	4.2	4.0
$V^{(d=0)}_1+V^{(d=0)}_2+V^{(d=2)}_3$	4.1	3.9
$V^{(d=0)}_1+V^{(d=0)}_2+V^{(d=3)}_3$	4.6	4.0
$V^{(d=1)}_1+V^{(d=0)}_2+V^{(d=0)}_3$	4.1	4.1
$V^{(d=1)}_1+V^{(d=0)}_2+V^{(d=1)}_3$	3.8	3.7
$V^{(d=1)}_1+V^{(d=0)}_2+V^{(d=2)}_3$	3.7	3.7
$V^{(d=1)}_1+V^{(d=0)}_2+V^{(d=3)}_3$	3.9	3.7
$V^{(d=2)}_1+V^{(d=0)}_2+V^{(d=1)}_3$	3.7	3.6
$V^{(d=2)}_1+V^{(d=0)}_2+V^{(d=2)}_3$	4.1	3.6
$V^{(d=2)}_1+V^{(d=0)}_2+V^{(d=3)}_3$	4.5	3.7
$V^{(d=3)}_1+V^{(d=0)}_2+V^{(d=1)}_3$	4.2	3.7
$V^{(d=3)}_1+V^{(d=0)}_2+V^{(d=2)}_3$	4.7	3.8
$V^{(d=3)}_1+V^{(d=0)}_2+V^{(d=3)}_3$	5.2	3.8
$V^{(d=3)}_1+V^{(d=0)}_2+V^{0.1}_3$	5.5	4.9
$V^{(d=3)}_1+V^{(d=0)}_2+V^{0.2}_3$	5.5	4.9
$V^{0.1}_1+V^{(d=0)}_2+V^{(d=0)}_3$	5.2	4.4
$V^{0.1}_1+V^{(d=0)}_2+V^{(d=1)}_3$	4.7	4.0
$V^{0.1}_1+V^{(d=0)}_2+V^{(d=2)}_3$	4.3	3.8
$V^{0.1}_1+V^{(d=0)}_2+V^{(d=3)}_3$	4.2	3.7
$V^{0.2}_1+V^{(d=0)}_2+V^{(d=0)}_3$	5.5	4.5
$V^{0.2}_1+V^{(d=0)}_2+V^{(d=1)}_3$	4.9	4.1
$V^{0.2}_1+V^{(d=0)}_2+V^{(d=2)}_3$	4.5	3.8
$V^{0.2}_1+V^{(d=0)}_2+V^{(d=3)}_3$	4.3	3.8
$V^{0.3}_1+V^{(d=0)}_2+V^{(d=0)}_3$	5.7	4.6
$V^{0.3}_1+V^{(d=0)}_2+V^{(d=1)}_3$	5.0	4.1
$V^{0.3}_1+V^{(d=0)}_2+V^{(d=2)}_3$	4.7	3.9
$V^{0.3}_1+V^{(d=0)}_2+V^{(d=3)}_3$	4.5	3.8

In our simulations, we compute the derivatives of the energy of the Hamiltonian, as given in Eq. (3), with respect to the LECs $C_{1}$, $C_{2}$, and $C_{3}$, that is $\partial \langle {E}\rangle /\partial C_{k}$. This approach allows us to determine the contributions from the *YNN* interactions as perturbative corrections,

26 $$\begin{aligned} \langle {E_{YNN}\rangle } = C_{1} \frac{\partial \langle {E}\rangle }{\partial C_{1}} +C_{2} \frac{\partial \langle {E}\rangle }{\partial C_{2}} +C_{3} \frac{\partial \langle {E}\rangle }{\partial C_{3}}. \end{aligned}$$

To determine the LECs $C_{1}$, $C_{2}$, and $C_{3}$, we perform standard least-squares fitting constrained by the separation energies. The results for the decuplet saturation scenario with one additional parameter $C_{2}$ are listed in Table 8. The complete set of *YNN* interactions without any constraints is provided in Table 9. For clarity, the five combinations that result in the smallest RMSD are highlighted in the main text in Tables 3 and 4.

## $^3_\varLambda$H infinite volume extrapolation

The infinite volume extrapolation of the $^3_\varLambda$H energies from a simplified interaction is presented in the main text, and in this section we discuss the infinite volume extrapolation of the $^3_\varLambda$H energies using a high-fidelity interaction.

We compute the ground state energies of the $^3_\varLambda$H system using the Hamiltonian $H_\mathrm{N^3LO} + H^{Y}_\textrm{free} + V_{Y N}$ through lattice Monte Carlo simulations in box sizes from $L=8$ to $L=15$. In addition, we compute the deuteron binding energies using the Hamiltonian $H_\mathrm{N^3LO}$ with exact diagonalization methods.

We perform infinite volume extrapolations for both the individual ground state energies and the $\varLambda$ separation energy simultaneously, employing the ansatz given in Eq. (11). The results are illustrated in Fig. 5. The main figure shows the individual energy extrapolations, while the inset highlights the separation energies.

As shown in Tables 5 and 6, the impact of the decuplet-saturated *YNN* interactions is below 1 keV, while the total contribution of the *YNN* interactions is approximately 42 keV. To improve the accuracy of these calculations, we plan to employ exact diagonalization methods for three-body and four-body hypernuclei in our future work.

## Splitting between $^4_\varLambda \text {H}^{0^+}$ and $^4_\varLambda \text {H}^{1^+}$

The splitting between $^4_\varLambda \text {H}^{0^+}$ and $^4_\varLambda \text {H}^{1^+}$, as well as the separation between $^4_\varLambda \text {He}^{0^+}$ and $^4_\varLambda \text {He}^{1^+}$, was initially measured with high precision using $\gamma$ spectroscopy [86]. These measurements indicated only a small charge symmetry breaking effect. More recent and precise measurements of the $^4_\varLambda \text {He}$ system [87] have refined our understanding of this charge symmetry effect. Given that our calculations do not resolve this difference, we use the averaged value for this splitting, $1.247 \pm 0.010$ MeV.

Using degenerate perturbation theory, we access the splitting between the ground and excited states in the four-body system. This method allows us to extract the splitting between these two states more precisely than what is reported in the main results section, as the uncertainties from the nuclear part drop out when focusing solely on the spin-breaking *YN* interaction.

Figure 6 shows the contribution of the spin-dependent part of the YN interaction to these two states when the Euclidean time extrapolation of the spin-dependent two-body *YN* force is performed independently from the nuclear part. Since this is the only spin-dependent part on the two-body level, the difference in the contributions result in the splitting of $B_\varLambda ( ^4_\varLambda \text {H}^{0^+})$-$B_\varLambda ( ^4_\varLambda \text {H}^{1^+}) = 0.7465 \pm 0.0035$ MeV of the $0^+$ and $1^+$ state. The relatively large difference of approximately  130 keV, compared to the results presented in Tables 5 and 6, is due to the dominant influence of the nuclear part during the Euclidean time extrapolation of the main result.

## Footnotes

1 We use this simplified interaction to speed up the fitting procedure. The extrapolated result of the N$^3$LO interaction is fully compatible with this result.

2 Since the fit is done only for light nuclei, this new freedom might increase the overall RMSD.

## Code Availability

Code/software will be made available on reasonable request. [Author’s comment: The code/software generated during and/or analysed during the current study is available from the corresponding author on reasonable request.]
